# Nature-Based Learning and Autism: A Systematic Review of Autistic Children’s Emotional Health and Behavioural Outcomes

**DOI:** 10.1192/bjo.2025.10207

**Published:** 2025-06-20

**Authors:** Emily Odame-Asante, Cornelius Ani, Salvatore Mura

**Affiliations:** SABP, Surrey, United Kingdom

## Abstract

**Aims:** Autism Spectrum Disorder (ASD) is a complex neurodevelopmental condition that affects social interaction, communication, and behaviour. Many children with ASD experience emotional dysregulation, heightened anxiety, and challenges in mainstream educational settings. Nature-based learning (NBL), including forest schools and outdoor education, has been proposed as an alternative approach that may support the emotional well-being and behavioural outcomes of autistic children. This systematic review examines the impact of NBL on children with ASD, focusing on emotional health, behavioural changes, and educational engagement.

**Methods:** A systematic search was conducted across four databases (PsycINFO, CINAHL, PubMed, and Embase) to identify primary studies examining the effects of NBL on autistic children. Additional sources, including grey literature and reference lists, were screened. Studies were included if they assessed behavioural, emotional, and educational outcomes in children under 18 years old diagnosed with ASD. Data were extracted and synthesized narratively to identify common themes.

**Results:** Eight studies met the inclusion criteria, comprising qualitative, quantitative, and mixed-methods research. Findings indicated that participation in NBL was associated with improvements in emotional regulation, reduced anxiety, enhanced social interactions, and increased engagement in learning activities. Some studies also reported positive effects on attendance and independence. Parents and educators perceived NBL as beneficial, although concerns were noted regarding disruption or routines and challenges with implementation.

**Conclusion:** Nature-based learning appears to offer significant benefits for children with ASD, particularly in supporting emotional well-being and social development. However, variations in study methodologies and small sample sizes highlight the need for further large-scale research. Future studies should explore standardized outcome measures, long-term impacts, and strategies for integrating NBL into educational provisions, ensuring tailored support for children with diverse needs.

